# Surface roughness and wear resistance of ormocer- versus methacrylate-based single-shade resin composites after simulated tooth brushing

**DOI:** 10.1186/s12903-026-08127-7

**Published:** 2026-04-02

**Authors:** Maha M. Ebaya, Mohamed Elshirbeny Elawsya

**Affiliations:** 1 Department of Conservative Dentistry, Faculty of Dentistry, Mansoura University, P.O. Box 35516, Algomhoria Street, Mansoura, Aldakhlia Egypt; 2 Faculty of Dentistry, New-Mansoura University, New-Mansoura City, Egypt; 3 Faculty of Dentistry, Mansoura National University, Gamasa, Egypt

**Keywords:** Simulated tooth brushing, Surface roughness, Single-shade composite, Wear resistance

## Abstract

**Background:**

To evaluate and compare the surface roughness and wear resistance of ormocer-based and methacrylate-based single-shade resin composites after simulated tooth brushing.

**Methods:**

Two single-shade composites; an ormocer-based composite (Admira Fusion X-tra, Voco) and a methacrylate-based composite (Omnichroma, Tokuyama), were used in this study. From each material, 20 disc-shaped specimens (3-mm thickness, 5-mm diameter) were prepared (*n* = 20). Specimens were subjected to 100,000 cycles of tooth brushing with a Colgate Max Fresh dentifrice (Colgate-Palmolive). The surface roughness was assessed before and after tooth brushing, and the changes in weight were determined after tooth brushing. Additional two specimens from each composite underwent scanning electron microscope (SEM) imaging. Paired and unpaired Student’s *t*-tests were used for statistical analysis (*p* < 0.05).

**Results:**

Surface roughness of both tested composites increased significantly after brushing (*p* = 0.002 for ormocer-based composite and *p* = 0.006 for methacrylate-based composite). However, there was no statistically significant difference among the two tested composites regarding surface roughness before brushing (*p* = 0.928) or after brushing (*p* = 0.696). The weights of both tested composites decreased significantly after brushing (*p* = 0.001), and the amount of methacrylate-based composite wear was significantly higher than that of the ormocer-based composite (*p* < 0.001).

**Conclusion:**

After simulated tooth brushing, both tested composites (ormocer-based and methacrylate-based) became rougher, whereas the ormocer-based composite exhibited greater wear resistance than did the methacrylate-based composite.

## Introduction

Resin composites are nowadays being used frequently for anterior teeth, with regard to its outstanding mechanical qualities, satisfactory esthetics, and preservation of tooth structure [1]. Filler and organic matrices have improved as a result of technological advancements in resin composite development. In recent years, fillers have changed, particularly in terms of particle type, size and distribution, which has enhanced the optical and mechanical qualities of dental composite [2, 3].

Additionally, monomer and photo-initiator progression enhance the mechanical characteristics of the resulting adhesive layer as well as the polymerization reactivity [4]. The two main monomers that are now widely used for the majority of dental composites are bisphenol A-glycidl methacrylate (Bis-GMA) and urethane dimethacrylate (UDMA). Its high viscosity necessitates the addition of low-molecular-weight monomers in order to attain an appropriate viscosity for the clinically employed final formulation. These diluent monomers increase the resin composite’s polymerization shrinkage, water sorption, and discoloration. In an effort to improve the qualities of composite restorative materials, new monomers have been studied as Bis-GMA-free composites [5].

The abbreviation for organically modified ceramic is ormocer. A molecule with a long-chain inorganic silica backbone and lateral organic chains is generated by hydrolysis as well as poly-condensation processes (sol–gel processing) [6]. Considering a high densely cross-linked polymer network emerges, the composite containing ormocer is claimed to have enhanced surface hardness, color stability, toughness, reduced polymerization shrinkage, and a higher degree of conversion. Since methacrylate groups have stronger chemical bonds, there have less free unreacted monomers in the polymer network, which further improves biocompatibility [7, 8].

Over the years, resin composites have undergone modifications that have improved their optical features and expanded their selection of opaque and translucent hues [9]. The capacity of restorative material to change its color toward the color of the surrounding hard dental tissues is known as the blending or chameleon effect. This reduces the number of shade guide tabs and prevent some degree of color mismatches [10]. Simplifying color matching begins with group-shade composite materials and continues until single-shade composites develops that is claimed to match various teeth shades [11].

Tooth brushing with varying types of toothpastes is considered the most common habit utilized by many individuals to improve the oral hygiene. Regardless of the kind of brush, stain and plaque removal are improved by both power-powered and manually driven brushes, such as oscillating brushes that run on batteries, rechargeable sonic effects, and rechargeable oscillating brushes [12, 13]. In addition to the mechanical and chemical characteristics of the tooth paste, various studies have linked the cleaning impact of regularly brushing to the mechanical motion of the brush itself [14, 15]. Despite the beneficial effects of cleaning your teeth, some dental restorations may have adverse effects, including surface deterioration and wear. In addition to, tooth surface erosion, and dental hypersensitivity [12, 16–18].

However, there is a lack of studies that have evaluated the surface changes of ormocer- and methacrylate-based single-shade composites [19]. Therefore, this study was designed as a controlled comparative analysis to evaluate the long-term surface performance of two major classes of contemporary resin composites. The materials were selected as canonical representatives within the clinically relevant category of single-shade resin composites. Admira Fusion X-tra (Voco) is the principal and most widely investigated single-shade composite based on ormocer chemistry. Omnichroma (Tokuyama) is a leading methacrylate-based single-shade composite utilizing a uniform supra-nano spherical filler technology. This focused pairing allows for a direct evaluation of how fundamental matrix chemistry (ormocer vs. methacrylate) influences durability under abrasive challenge.

While previous research has examined composite wear, there is a paucity of direct, long-term comparative data on modern single-shade composites with fundamentally different matrix chemistries. Specifically, a controlled comparison of the surface durability of an ormocer-based composite versus a methacrylate-based composite under a clinically relevant, extended brushing simulation is lacking. Therefore, the current study aimed to conduct such a comparison by evaluating and comparing the surface roughness and wear resistance of these two material classes after long-term simulated tooth brushing abrasion. The first null hypothesis tested was that there would be no significant differences in surface roughness before and after simulated tooth brushing for the tested composites. The second null hypothesis tested was that there would be no significant differences in wear resistance between the tested composites after simulated tooth brushing.

## Materials and methods

### Materials

Two single-shade composites; an ormocer-based composite (Admira Fusion X-tra, Voco) and a methacrylate-based composite (Omnichroma, Tokuyama), were utilized in this study. Materials used in this study are presented in Table 1.

**Table 1:** Materials used in the study

Material	Manufacturer	Type	Composition	Lot No.
Admira fusion X-tra	VOCO GmbH, Cuxhaven, Germany	Nano-hybrid ormocer- based composite	Matrix: Resin ormocer Filler: Silicon oxide nano-filler, Glass ceramics filler (1 μm) Filler content: 84 (w/w)	1,604,218
Omnichroma	Tokuyama Dental, Tokyo, Japan	Supra-nano filled composite	UDMA, TEGDMA, Mequniol, BHT and UV absorber. Filler content: 79 (w/w) of spherical silica-zirconia filler (Mean particle size: 0.3 μm)	1,602,201

### Methods

#### Sample size calculation

Sample size was calculated based on previous research [19]. Using G*Power program version 3.1.9.7 to calculate sample size based on effect size of 1.195, using 2-tailed test, α error = 0.05 and power = 95%, the calculated sample size was 20 in each group (*n* = 20).

#### Study design and specimens’ preparation

**Fig. 1:**
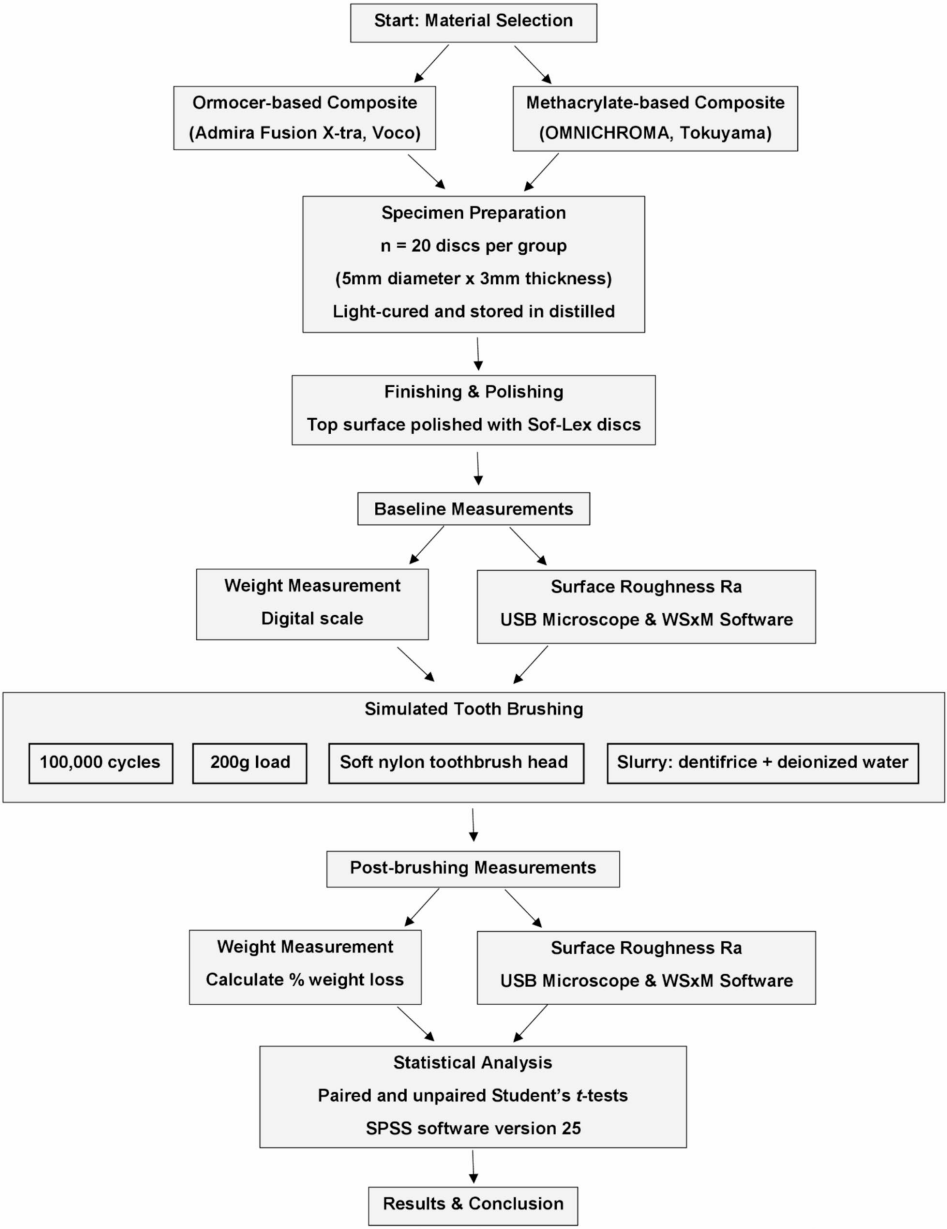
A diagram showing the study design and research steps

The study was presented to and approved by The Dental Research Ethics Committee under protocol number A0102024CD (Faculty of Dentistry, Mansoura University). Twenty specimens (*n* = 20) were prepared for each group (ormocer-based composite group and methacrylate-based composite group). A split Teflon mold was constructed to allow the fabrication of standard-sized disc-shaped specimens with a 3 mm thickness and 5 mm diameter [20]. A Mylar strip was placed over a glass slide, and then the Teflon mold was placed on it. Each material was correctly adapted via a modeling instrument for resin composite (CompoRoller, Kerr, Switzerland) assuring perfect adaptation with the mold until it was slightly overfilled, and then another Mylar strip and glass slide were placed over the mold to cover its top surface. To guarantee uniform stress distribution and standardize the pressure applied to each specimen, a 500 g calibration weight was set on the glass slide for 20 s. Each specimen was subsequently cured for 20 s from the top surface using a light-emitting diode (LED) device with an intensity of 1400 mW/cm^2^ (Radii Xpert, SDI Limited, Bayswater Victoria, Australia), then the bottom surface was cured in the same manner. The light curing device intensity was regularly checked with a radiometer (Apoza Enterprise, Chung-Cheng RD, Taiwan). After polymerization, the specimens were removed from the mold and stored in distilled water on a dark container in an incubator (DS20, BioStep, Egypt) at a temperature of 37 ± 1 °C for 24 h; simulating the oral cavity conditions, to enable post-polymerization and the elution of unreacted components. Study design and research steps are presented in Fig. 1.

#### Finishing and polishing

The top surfaces of all specimens (at where simulated tooth brushing would be performed and surface roughness would be measured) were polished. Polishing was accomplished using aluminum oxide discs (Sof-Lex, 3 M ESPE, 44-0007-7442-0-A lot N664515, St Paul, MN, USA). The medium, fine, and superfine grits were used in that order. The polishing procedure was performed by a single trained operator using a low-speed handpiece at 12,000 rpm mounted in a custom jig to maintain a constant pressure of 2 N (verified by a digital scale). A metronome-guided, unidirectional stroking pattern (30 strokes per grit) was used to ensure consistency. New Sof-Lex discs were used to polish each specimen. Following polishing, each specimen was cleaned in an ultrasonic water bath for 5 min (Ultrasonic cleaner L & R 2014, Kearny, NJ, USA). The specimens were kept at 37 ± 1 °C in distilled water.

#### Weight and surface roughness measurements

**Fig. 2:**
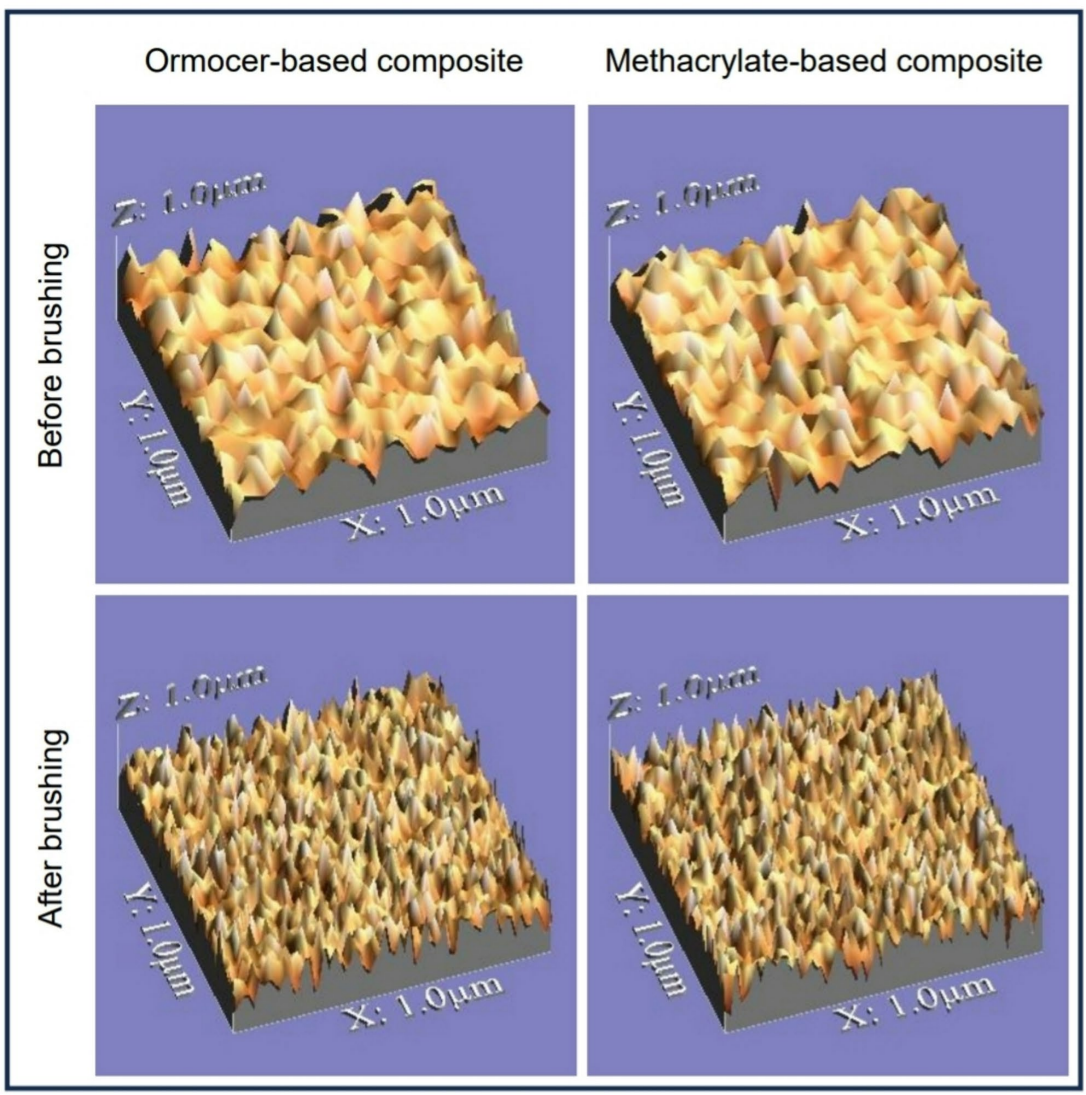
Surface roughness images of representative specimens from all tested groups

To establish a stable baseline weight and account for water sorption, specimens were weighed daily over 14 days following water storage until a constant mass was achieved on five consecutive days. This stable weight was recorded as the pre-brushing baseline [20]. An electric digital scale (AG245 Metter, Switzerland) was used to assess the weight of each specimen. In addition, the specimens were evaluated and photographed via a USB digital microscope (Capture Digital Microscope, Guangdong, China). A fixed magnification of 120X with a 0.8 mm cut off and 2.4 mm evaluation length was applied to the images, which were captured at highest resolution and connected to a computer compatible with IBM [21]. When they were captured, each image had a resolution of 1280 × 1024 pixels. Using a Microsoft Office Picture Manager, the images were cropped to 350 × 400 pixels. After analysis using WSxM software, a 3D picture of the specimens’ surface was produced. For each specimen, three 3D pictures were captured, each covering an area of 10 μm × 10 μm. To analyze average surface roughness (Ra) in µm, WSxM software was used [22–24]. The surface roughness images of representative specimens from all the tested groups are presented in Fig. 2.

#### Tooth brushing wear simulation

The rehydration process mimicked the initial day of restoration placement in the oral environment. Three-body simulated tooth brushing wear testing was carried out via programmable logic-controlled device; it had four-chamber multimodal dual-axis ROBOTA (Model ACH-09075DC-T, AdTech Technology Co., Germany). Simulating both vertical and horizontal movements concurrently was made possible by the device. Each chamber has a lower plastic specimen holder where the specimen can be anchored and an above Jackob’s chuck that can be screwed into place as a toothbrush antagonist holder. In the lower specimen holder, the discs were placed in Teflon housing protruding 0.5 mm from the surface. A weight of 200 g, which was comparable to 2 N of brushing force was exerted [20, 25]. All wear test parameters are presented in Table 2.

**Table 2:** Wear test parameters used in the study

Vertical movement: 1 mm	Horizontal movement: 3 mm
Forward speed: 90 mm/s	Backward speed: 40 mm/s
Rising speed: 90 mm/s	Descending speed: 40 mm/s
Cycle frequency: 1.6 Hz	Weight per specimen: 200 g
Torque: 2.4 N.m	

##### - Toothbrush and abrasive medium

Soft nylon bristle toothbrush heads (Oral B Indicator; Procter & Gamble Nanning, Kwangsi, China) were used with a slurry which was prepared by mixing a 2:1 ratio of deionized water and a Colgate Max Fresh dentifrice (Colgate-Palmolive) with RDA 150–200. The slurry was added to the tooth brushing machine’s station and changed every four new specimens. Changes were made to the toothbrush heads after 5000 cycles. The discs were subjected to a revolution of 100,000 cycles simulating a long-term clinical service, equivalent to approximately 10 years of twice-daily brushing [26]. The specimens were cleaned after testing with running water and then placed in an ultrasonic bath for 10 min prior to measurement. The weight and surface roughness of the abraded specimens were determined again and recorded as described previously. Wear measurements were expressed as a weight loss percentage.

### Scanning electron microscopy (SEM) analysis

Two representative specimens from each composite underwent surface morphological analysis using scanning electron microscope (SEM). Specimens were evaluated at baseline and following the completion of the brushing simulation. The specimens were cleaned in an ultrasonic bath, air-dried, and sputter-coated with a thin layer of gold to ensure conductivity. The examination was performed using a scanning electron microscope (JSM-6510LV, JEOL Ltd., Japan) operating at an accelerating voltage of 30 kV under high vacuum. Micrographs of the central region of each specimen were captured at standard magnifications of 1000x, 2000x, and 3000x to assess surface topography, wear patterns, and the integrity of the filler-matrix interface.

### Statistical analysis

SPSS^®^ software version 25 (SPSS Inc., Chicago, IL, USA) was used for data analysis. The data distribution’s normality was evaluated using Shapiro-Wilk test. The data were normally distributed and parametric. Consequently, descriptive statistics are presented as the means and standard deviations. Paired and unpaired Student’s *t*-tests were used to compare the mean values of parametric data between the two groups. The *p*-value < 0.05 were regarded as statistically significant.

## Results

### Surface roughness results

**Fig. 3:**
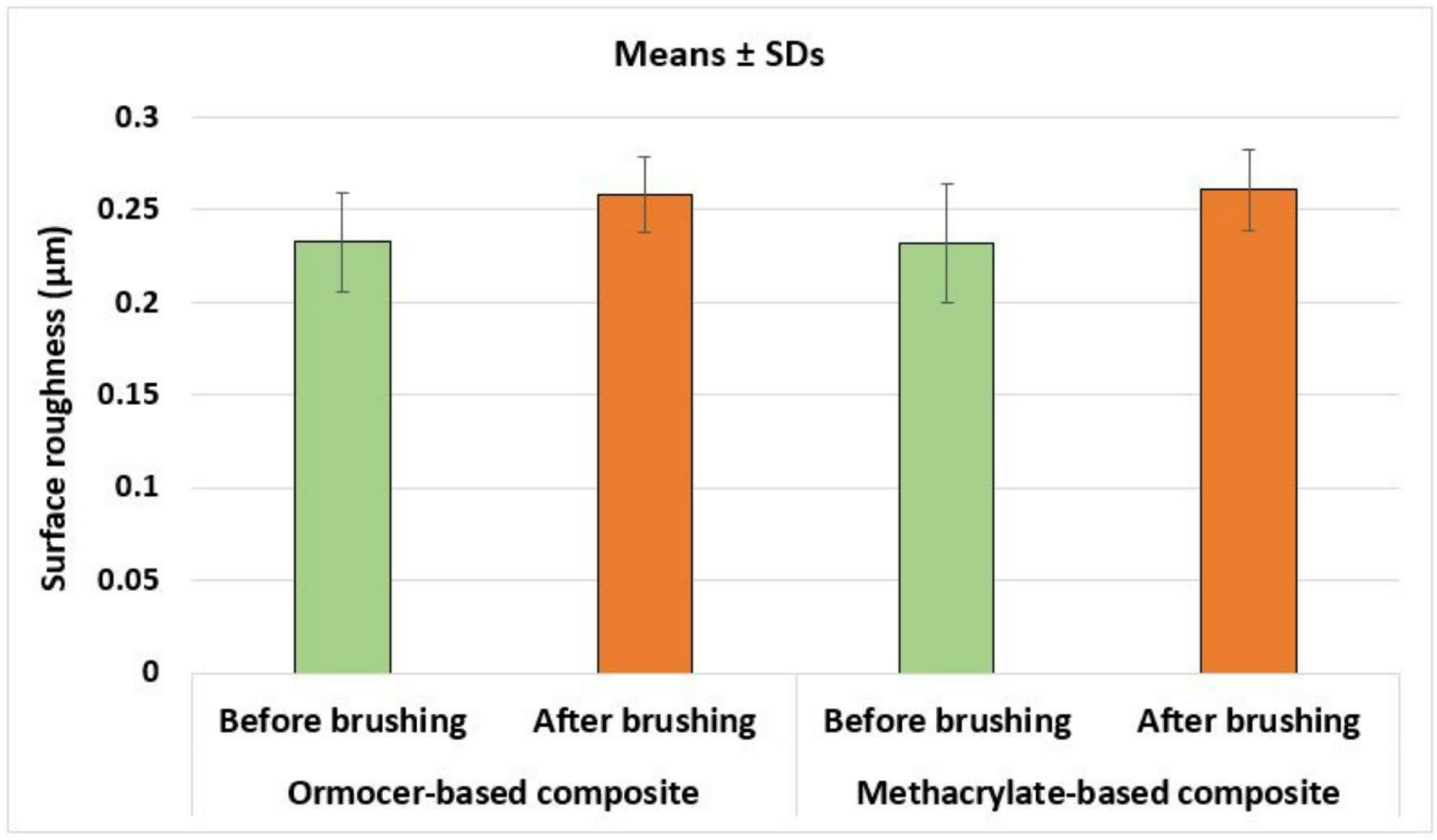
A bar chart represents means ± SDs of surface roughness values (µm) of the two tested composites before and after simulated tooth brushing

The surface roughness results revealed that surface roughness of both tested composite materials increased significantly after brushing (*p* = 0.002 for ormocer-based composite and *p* = 0.006 for methacrylate-based composite). However, there was no statistically significant difference between the two tested composite materials regarding surface roughness before brushing (*p* = 0.928) or after brushing (*p* = 0.696). The surface roughness mean values and standard deviations of both tested composites before and after brushing are presented in Table 3; Fig. 3.

**Table 3:** Comparison of surface roughness (µm) between the two tested composites before and after simulated tooth brushing

	Surface roughness before brushing	Surface roughness after brushing	Paired student’s t-test *p*-value
Ormocer-based composite	0.2327 ± 0.0267	0.2580 ± 0.0208	0.002*
Methacrylate-based composite	0.2318 ± 0.0324	0.2606 ± 0.0218	0.006*
Unpaired student’s *t*-test *p*-value	0.928	0.696	

Mean values ± SDs

*p is significant at 5% level

### Wear resistance results

The weight of both tested composites decreased significantly after brushing (*p* = 0.001), and the amount of methacrylate-based composite wear was significantly higher than that of the ormocer-based composite (*p* < 0.001). The mean values and standard deviations of weight before and after brushing and percentage weight loss for both tested composites are presented in Table 4.

**Table 4:** Comparison between the weights (g) before and after simulated tooth brushing and percentage weight loss of the two tested composites

	Weight before brushing	Weight after brushing	Paired student’s t-test *p*-value	Percentage weight loss
Ormocer-based composite	0.13528 ± 0.00281	0.13480 ± 0.00246	0.001*	0.35465 ± 0.06007
Methacrylate-based composite	0.12818 ± 0.00290	0.12760 ± 0.00286	0.001*	0.46015 ± 0.08300
Unpaired student’s *t*-test *p*-value				0.000*

Mean values ± SDs

*p is significant at 5% level

### SEM evaluation

**Fig. 4:**
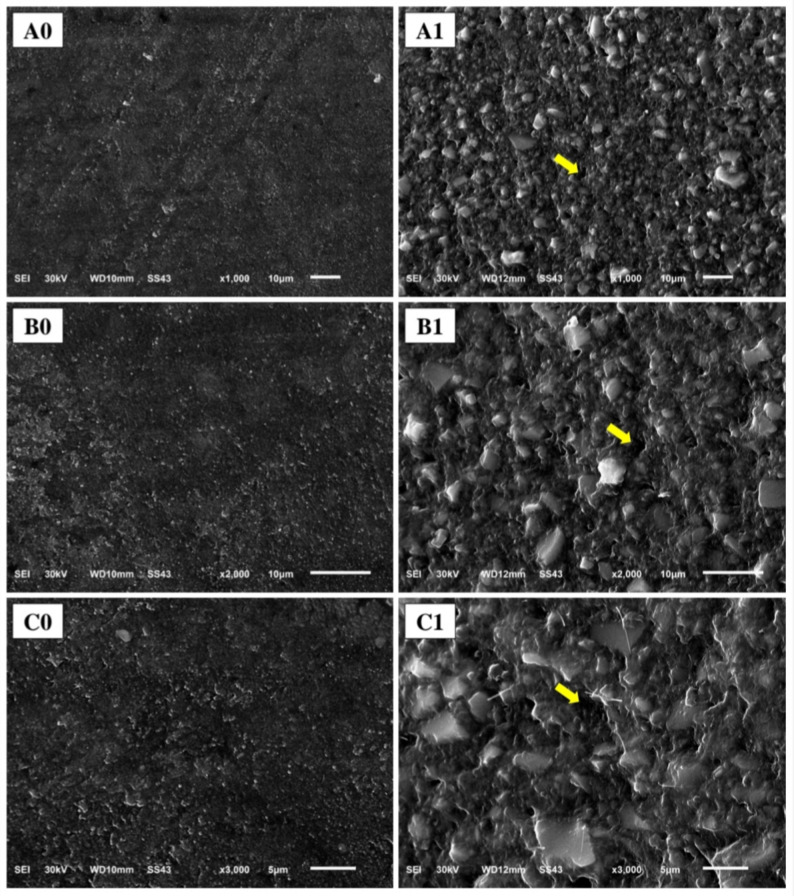
Representative SEM micrographs of ormocer-based composite at 1000x, 2000x, and 3000x magnifications (**A**, **B**, and **C** respectively). **0**: before brushing, **1**: after brushing. Arrows showing filler particles detachment

**Fig. 5:**
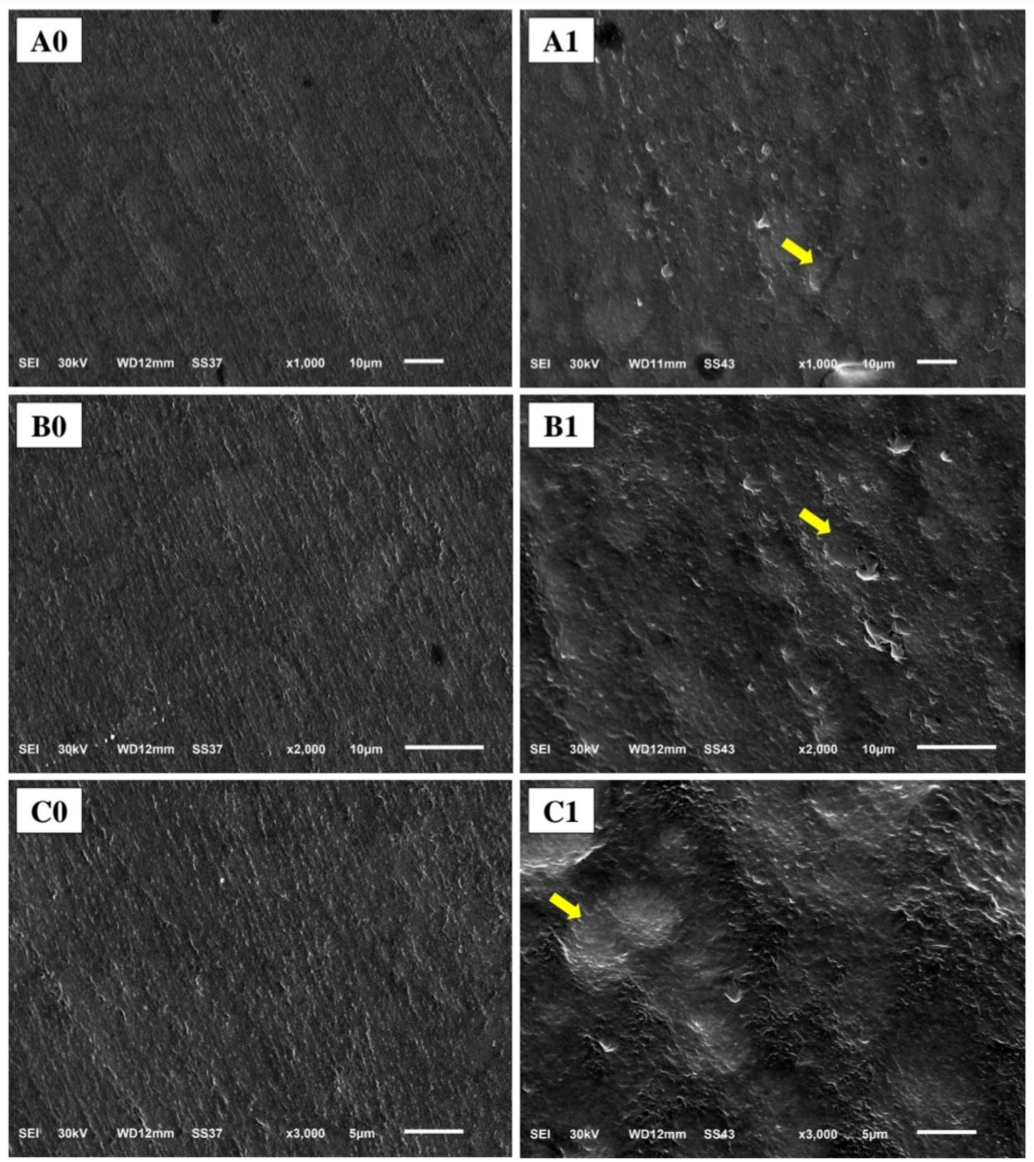
Representative SEM micrographs of methacrylate-based composite at 1000x, 2000x, and 3000x magnifications (**A**, **B**, and **C** respectively). **0**: before brushing, **1**: after brushing. Arrows showing recessed areas of resin matrix

Representative SEM micrographs of ormocer-based composite (Fig. 4), and methacrylate-based composite (Fig. 5) revealed distinct surface morphologies before and after abrasion. After 100,000 brushing cycles, the surfaces of both composites revealed distinct morphological signatures of wear. The ormocer-based composite surface (Admira Fusion X-tra) displayed a non-directional, particulate texture where filler particles appeared prominent and features suggested filler particles detachment. The methacrylate-based composite surface (Omnichroma) exhibited a uniform pattern of parallel linear grooves with recessed areas of resin matrix, consistent with a differing mode of surface alteration.

## Discussion

The present study addressed a specific gap by providing a direct, long-term comparison of the surface durability of two major classes of single-shade composites. The significant difference in wear resistance observed under long-term simulated brushing suggests that, even within this clinically convenient category, the fundamental choice of matrix chemistry can have a substantial impact on a material’s durability. The first null hypothesis stated that there would be no significant difference in surface roughness before and after simulated tooth brushing for the two tested composites was rejected. Additionally, the second null hypothesis stated that there would be no significant difference in wear resistance between the two tested composites after simulated tooth brushing was also rejected.

Among the crucial characteristics of dental composite restorations are being polishable to a glossy, smooth surface, simulating that of dental enamel, and preserving surface quality throughout time in spite of continuous abrasive exposure, such as tooth brushing. Tooth brushing is an essential factor that influences clinical performance and occlusion; thus, the durability of the restorations is affected by the presence of areas that cause plaque retention, gingival irritation, staining and/or recurrent caries. Several studies have shown that surface roughness and wear significantly increase after tooth brushing [17, 20].

The present study employed an accelerated aging protocol of 100,000 brushing cycles to simulate a clinically significant, long-term service period. Based on established models equating laboratory brushing cycles to clinical time—where one cycle approximates one brushing event—this regimen corresponds to the abrasive challenge of approximately 10 years of twice-daily oral hygiene [26]. This extended simulation is critical for a comparative study of this nature, as it moves beyond assessing initial material properties to evaluate long-term durability.

Accordingly, the specimens in the current study were finished and polished before the testing in an attempt to replicate clinical circumstances [27]. The identical finishing and polishing procedure was used to each specimen. The most common approach was the Sof-lex polishing disc series. Because aluminum oxide discs can create polished, nondestructive surfaces that are smooth and less prone to wear, they have been proposed as a standard procedure [28].

The mechanisms underlying the wear of composites are complicated and insufficiently understood. The loss of material constituents that leads to the loss of anatomic form is called wear. In this study, a tooth brushing simulation was used to examine the wear of ormocer-based and methacrylate-based composites. Wear of the resin (organic portion) could be explained as the abrasion mechanism of the resin composites, leading to the formation of gaps that vary in the size of the filler, thus increasing its roughness [29]. In earlier times, the most important factor for producing resin composites with greater resistance was filler qualities. Nonetheless, silane bonding agent and organic matrix composition are also crucial elements involved in improving the mechanical characteristics of resin composite [20, 30].

Regarding the assessment of surface roughness, the ormocer-based and methacrylate-based composites were considered to be clinically acceptable in respect to bacterial adhesion and patient comfort [19]. These outcomes may be attributable to the sol-gel technique used to create the nanoparticles in both materials. This process results in the formation of nanospheres with highly smooth polished surfaces [17]. In addition, considering the methacrylate-based composite (Omnichroma), its surface roughness must be considered in light of its unique filler technology. It contains uniformly sized, spherical supra-nano fillers designed to create a densely packed structure. Moreover, fillers were based on their own patented “Sub-Micro-Pearl-Technology”. This feature results in a highly smooth polished surface [19, 24].

The result of surface roughness in the current study is consistent with Gurbuz et al. [31] and Cunha et al. [32] who found that ormocer-based composites exhibited no significant difference regarding surface roughness compared to conventional composites due to their analogous filler load and size. In contrast, Tagtekin et al. [33] stated that since the filler particles in ormocer are tougher than those in the matrix, they exhibit preferential loss during finishing and polishing, leaving the filler phase on a positive surface and increasing surface roughness.

The surface roughness of the specimens increased after tooth brushing, but the difference was not significant, which contradicted the findings of a number of earlier investigations. Ishikiriama et al. [17], Han et al. [34], Al Khuraif [35], and de Moraes et al. [36] evaluated surface roughness of ormocer and conventional resin composites and reported that, after tooth brushing the surface roughness of the specimens did not significantly differ due to the surface fillers becoming exposed after the resin matrix has been abraded. Additionally, the matrix’s absorption of water raises the osmotic pressure at the interface between matrix phase and fillers phase, which causes surface cracks and the hydrolytic breakdown of silane, which causes the filler to separate from the surface and form tiny holes that raise the surface roughness [37].

In this way, simulated tooth brushing over time induced abrasive wear on the surfaces of the restorations. The extent of this wear is depending upon many factors, such as tooth brushing habits, the abrasiveness of the employed dentifrices, the consistency of the toothbrush bristles, and other characteristics related to the restorative material [34]. The dentifrice’s properties are affected by the type and size of the abrasive and the ratio of dentifrice to water; additionally, tooth brushing is influenced by the number, rigidity, and configuration of the tufts and bristles. Nevertheless, since all parameters mentioned were consistent for all the groups in the present study, abrasion resistance of the materials appears to be determined by the properties inherent to each one [29].

According to the wear values, the ormocer-based composite was exhibited reduced susceptibility to wear following tooth brushing simulation. To improve composites performance, manufacturers have created composites with different filler sizes (ranging from 5 to 100 nm) and distributions. These materials’ high filler load, which results from the fillers’ small size, is responsible for their mechanical properties, which include low abrasion, high flexural strength, resistance to fracture, and minimal polymerization shrinkage [38]. The average distance between neighboring particles was lower with smaller particles than with the coarsest filler particles, which could account for the increase in wear resistance [39]. The organic matrix is shielded from wear by this size and dispersion, which increases the ormocer-based composite’s longevity.

With respect to the methacrylate-based composite, the presence of the triethylene glycol dimethacrylate (TEGDMA) monomer can play a role in its wear rates. This monomer reduces the resin matrix’s viscosity and results in increased water sorption and susceptibility to hydrolysis, which increases the degree of wear of the material [40, 41]. These outcomes were consistent with those of Oliveira et al. [42], who reported that the use of an ormocer-based composite is relevant for resisting wearing in comparison with the performance of the methacrylate-based composite. However, this result contrast with those of Ishikiriama et al. [17], who reported that ormocer-based composites with a high filler percentage can be associated with increased wear. Mass loss from the surface of materials with high filler amount might result from weak bonding between the matrix and fillers and an increase in the coefficient of friction between the two substances [43].

The SEM micrographs provide direct visual evidence of the divergent wear mechanisms. The uniform pattern of parallel linear grooves with recessed areas of resin matrix on the methacrylate-based composite signifies preferential abrasive removal of the resin matrix. This process of generalized matrix loss directly accounts for the material’s significantly higher gravimetric wear. Furthermore, this abrasion creates a topography with deeper valleys, which is precisely what the non-contact profilometer detected as a trend toward a higher mean surface roughness (Ra), as the instrument’s measurement is based on vertical deviations. While the ormocer-based composite’s surface, characterized by a particulate texture, partial filler particles exposure, and filler particles detachment experiences a different alteration with the same impact on peak-to-valley depth and correlating with its superior wear resistance.

The primary aim was to determine which material is more resistant to wear under sustained, clinically relevant abrasive challenge. Our finding that the ormocer-based composite exhibited significantly lower material loss (weight) after this long-term test provides compelling in-vitro evidence for its superior wear resistance and potential for better anatomical form preservation over time compared to the methacrylate-based composite. While both materials remained within a clinically acceptable range for surface roughness, the differential wear behavior highlights how fundamental differences in matrix chemistry and filler technology can meaningfully impact long-term clinical performance.

This in-vitro study has certain limitations that outline clear pathways for future research. First, the comparative analysis was intentionally focused on two specific, commercially successful materials as leading representatives of the ormocer- and methacrylate-based composite classes within the single-shade category. While this controlled design allows for a direct evaluation of fundamental matrix chemistry, it limits the generalizability of the results to all materials within these broad families. Second, the surface finishing protocol employed a single, albeit common, multi-step aluminum oxide disc system. Polishing outcomes can vary with different instrument types (e.g., silicone polishers, diamond pastes). Additionally, the analytical framework did not include complementary mechanical assessments, such as microhardness testing, which could have provided valuable data on subsurface resin matrix stability and filler-matrix integrity following abrasion. Furthermore, while weight loss and surface roughness provide clear quantitative measures of wear and smoothness, complementary analyses such as 3D profilometry for volumetric loss or long-term chemical stability tests would offer even deeper mechanistic insight. Finally, the simulation was restricted to mechanical abrasion, excluding other intraoral aging factors like thermal cycling and pH fluctuations.

## Conclusion

After a long-term simulated tooth brushing regimen, both the ormocer-based composite (Admira Fusion X-tra) and the methacrylate-based composite (Omnichroma) exhibited a significant increase in surface roughness. However, the Admira Fusion X-tra composite demonstrated significantly greater wear resistance compared to Omnichroma. These findings suggest that within the category of single-shade composites, the fundamental ormocer matrix chemistry may offer an advantage in durability under sustained abrasive challenge, despite providing a comparable final surface smoothness to the methacrylate-based material.

## Data Availability

The data that support the findings of this study are available from the corresponding author upon reasonable request.

## Abbreviations

µm: Micrometer
3D: 3 Dimensions
BHT: Butylated hydroxytoluene
g: Gram
h: Hour
LED: Light emitting diode
min: Minute
N: Newton
RDA: Relative Dentin Abrasivity
Ra: Roughness average
s: Second
SEM: Scanning electron microscope
SDs: Standard deviations
Bis-GMA: Bisphenol A-glycidl methacrylate
TEGDMA: Triethylene glycol dimethacrylate
UDMA: Urethane di-methacrylate
UV: Ultraviolet
